# Butyrate, Forskolin, and Lactose Synergistically Enhance Disease Resistance by Inducing the Expression of the Genes Involved in Innate Host Defense and Barrier Function

**DOI:** 10.3390/antibiotics10101175

**Published:** 2021-09-27

**Authors:** Qing Yang, Melanie A. Whitmore, Kelsy Robinson, Wentao Lyu, Guolong Zhang

**Affiliations:** 1 Department of Animal and Food Sciences, Oklahoma State University, Stillwater, OK 74078, USA; qing.yang@okstate.edu (Q.Y.); melanie.whitmore@okstate.edu (M.A.W.); kelsy.robinson@okstate.edu (K.R.); lvwt@zaas.ac.cn (W.L.); 2 Poultry Production and Product Safety Research Unit, USDA–Agricultural Research Service, Fayetteville, AR 72701, USA; 3 State Key Laboratory for Managing Biotic and Chemical Threats to the Quality and Safety of Agro-Products, Institute of Agro-Product Safety and Nutrition, Zhejiang Academy of Agricultural Sciences, Hangzhou 310021, China

**Keywords:** antimicrobial resistance, antibiotic alternatives, antimicrobial peptides, host defense peptides, butyrate, forskolin, lactose, necrotic enteritis, coccidiosis, poultry

## Abstract

The rising concern of antimicrobial resistance highlights a need for effective alternatives to antibiotics for livestock production. Butyrate, forskolin, and lactose are three natural products known to induce the synthesis of host defense peptides (HDP), which are a critical component of innate immunity. In this study, the synergy among butyrate, forskolin, and lactose in enhancing innate host defense, barrier function, and resistance to necrotic enteritis and coccidiosis was investigated. Our results indicated that the three compounds synergistically augmented the expressions of multiple HDP and barrier function genes in chicken HD11 macrophages. The compounds also showed an obvious synergy in promoting HDP gene expressions in chicken jejunal explants. Dietary supplementation of a combination of 1 g/kg sodium butyrate, 10 mg/kg forskolin-containing plant extract, and 10 g/kg lactose dramatically improved the survival of chickens from 39% to 94% (*p* < 0.001) in a co-infection model of necrotic enteritis. Furthermore, the three compounds largely reversed growth suppression, significantly alleviated intestinal lesions, and reduced colonization of *Clostridium perfringens* or *Eimeria maxima* in chickens with necrotic enteritis and coccidiosis (*p* < 0.01). Collectively, dietary supplementation of butyrate, forskolin, and lactose is a promising antibiotic alternative approach to disease control and prevention for poultry and possibly other livestock species.

## 1. Introduction

Necrotic enteritis (NE) is caused by the Gram-positive bacterium *Clostridium perfringens* [1], while the etiological agents of coccidiosis are *Eimeria*, a genus of apicomplexan parasites [2]. Both NE and coccidiosis are among the most economically significant infectious diseases facing the poultry industry causing growth retardation, morbidity, and even mortality [1,2]. With a growing number of countries having withdrawn in-feed antimicrobials for growth promotion and disease prophylactics [3], NE and coccidiosis are expected to become more prevalent and pose a greater threat to animal health and welfare and the sustainability of the poultry industry [4,5]. Therefore, there is an urgent need for the development of effective alternatives to antibiotics for livestock and poultry production [4,5,6].

Enhancing the production of endogenous host defense peptides (HDPs) is being actively explored as a promising antibiotic-free strategy for disease control and prevention [7,8,9,10]. HDPs, also known as antimicrobial peptides, are a group of small peptides produced mainly by mucosal epithelial cells and phagocytes [11,12,13,14]. As a critical component of the innate immune system [11,12], HDPs possess direct broad-spectrum antimicrobial activities against bacteria, fungi, protozoa, and viruses [11,12,14]. Moreover, HDPs are known to exert multifaceted regulatory influences on the conduct of immunity such as neutralization of endotoxin, chemotaxis of immune cells, activation of phagocytes, and initiation of adaptive immunity [12]. Additionally, HDPs are also capable of improving barrier function by stimulating the production of mucins and tight junction proteins [15]. Due to potent antimicrobial, immunomodulatory, and barrier-protective properties of HDPs, strategies to enhance HDP synthesis have been shown to protect the host against infections [7,8,9,10]. A variety of small-molecule compounds such as fatty acids, phytochemicals, and vitamin D_3_ have been shown to induce HDP expression and are being developed for antimicrobial therapies [7,8,9,10,16].

Butyrate is a major short-chain fatty acid produced by bacterial fermentation of dietary fibers in the intestine [17,18] and has been demonstrated to improve disease resistance of humans and animals through HDP stimulation [7,19,20]. Furthermore, butyrate improves epithelial barrier function by enhancing the synthesis of tight junction proteins and mucins [17,18]. Forskolin, a natural labdane diterpene present in the Indian *Coleus* plant (*Coleus forskohlii*) [21], improves HDP synthesis in both human and chicken cells [22,23]. Lactose, a disaccharide and a major component of milk, has been reported to induce human HDP production [24]. In addition, a synergy in HDP induction between butyrate and forskolin [23] and also between butyrate and lactose [24] have been demonstrated separately in chickens and humans, respectively.

However, a potential synergy among butyrate, forskolin, and lactose in HDP expression, barrier function, and disease resistance is yet to be explored. The aim of this study was, therefore, to explore a synergistic effect among these three natural products in enhancing innate immunity, mucosal barrier integrity, and protection of chickens against NE and coccidiosis. The expressions of HDP and barrier function genes in chicken HD11 macrophage cells and jejunal explants were evaluated in response to butyrate, forskolin, and lactose individually or in combinations. The three compounds were further supplemented in the diet for evaluating their efficacy in protecting broiler chickens against experimental NE and coccidiosis. Both of our in vitro and in vivo results demonstrated the potential of administrating a combination of butyrate, forskolin, and lactose as a novel alternative to antibiotics for disease control and prevention in poultry and possibly other livestock species as well.

## 2. Results

### 2.1. Synergistic Induction of Chicken HDP Gene Expression by Butyrate, Forskolin, and Lactose

**Figure 1:**
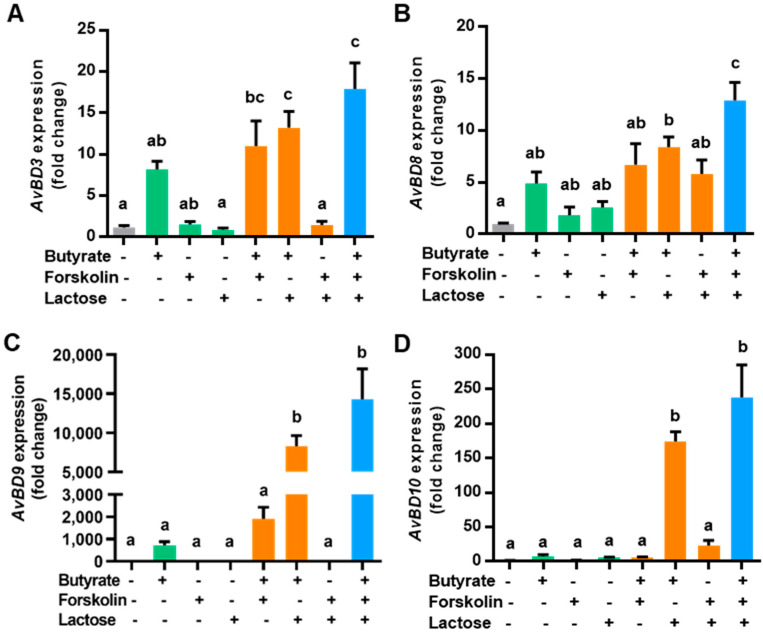
Induction of host defense peptide gene expression in macrophages by butyrate, forskolin, and lactose. Chicken HD11 macrophages were stimulated in triplicate with 2 mM butyrate, 5 μM forskolin, and 0.1 M lactose individually or in combinations of two or three compounds for 24 h, followed by RT-qPCR analysis of the gene expressions of avian β-defensin 3 (*AvBD3*) (**A**), *AvBD8* (**B**), *AvBD9* (**C**), and *AvBD10* (**D**). Results are shown as means ± SEM of three independent experiments. Means not sharing a common letter are significantly different (*p* < 0.05; one-way ANOVA and *post hoc* Tukey’s test).

To evaluate a potential synergy in HDP induction among butyrate, forskolin, and lactose, we stimulated chicken HD11 macrophages with the three compounds individually or in different combinations for 24 h, followed by quantitative reverse transcription PCR (RT-qPCR) analysis of the expressions of four representative HDP genes). Butyrate at 2 mM increased *AvBD3* expression by 8-fold, while 5 μM forskolin or 0.1 M lactose did not obviously improve *AvBD3* expression in HD11 cells (Figure 1A). Butyrate in combination with forskolin or lactose induced a higher level of *AvBD3* expression relative to butyrate alone, while the forskolin/lactose combination had no effect on *AvBD3* gene expression. Importantly, a combination of butyrate, forskolin, and lactose gave a 17-fold increase in *AvBD3* expression, higher than any two-compound combination (Figure 1A).

Similarly, 2 mM butyrate gave a modest induction of *AvBD8* (Figure 1B), *AvBD9* (Figure 1C), and *AvBD10* (Figure 1D), while forskolin or lactose alone had a minimum effect. Butyrate combined with forskolin or lactose showed an obvious synergy, whereas the forskolin/lactose combination largely had no impact. Importantly, the highest induction of all three HDP genes occurred when butyrate, forskolin, and lactose were used together (Figure 1B–D). It is noted that different HDP genes were apparently regulated at different magnitudes. While *AvBD8* was induced by 13-fold in response to the three-compound combination, *AvBD9* showed a more than 14,000-fold increase (Figure 1B,C). It is also noteworthy that several other HDP genes such as *AvBD1* were unaffected by any of the three compounds or their combinations (data not shown).

**Figure 2:**
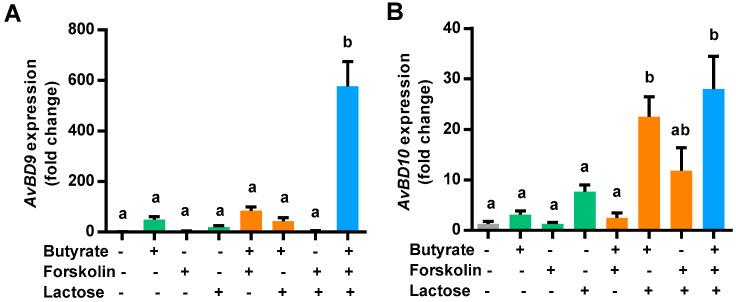
Induction of host defense peptide gene expression in chicken jejunal explants by butyrate, forskolin, and lactose. Chicken jejunal segments were cultured and treated in triplicate with 4 mM butyrate, 5 μM forskolin, and 0.1 M lactose individually or in different combinations for 24 h, followed by RT-qPCR analysis of the gene expressions of *AvBD9* (**A**) and *AvBD10* (**B**). Results are presented as means ± SEM of 3–4 independent experiments. Means not sharing a common letter are significantly different (*p* < 0.05; one-way ANOVA and *post hoc* Tukey’s test).

To confirm the synergy in HDP gene induction among butyrate, forskolin, and lactose ex vivo, we prepared chicken jejunal explants and analyzed the expressions of *AvBD9* and *AvBD10* following stimulation with the three compounds alone or in different combinations. Butyrate (4 mM) and 0.1 M lactose had a modest effect on the induction of both *AvBD9* (Figure 2A) and *AvBD10* (Figure 2B), while 5 µM forskolin showed little impact. The efficacy of two-compound combinations varied in modulating the expressions of *AvBD9* and *AvBD10*, while the strongest induction of both HDP genes was observed when all three compounds were applied. In the case of *AvBD9*, butyrate augmented *AvBD9* expression by approximately 50-fold, but a combination of butyrate, forskolin, and lactose gave a nearly 600-fold increase (Figure 2A). Collectively, these results indicated that butyrate, forskolin, and lactose are synergistic in enhancing the expressions of multiple HDP genes both in vitro and ex vivo.

### 2.2. Induction of Tight Junction Protein and Mucin 2 Gene Expressions by Butyrate, Forskolin, and Lactose in HD11 Cells

**Figure 3:**
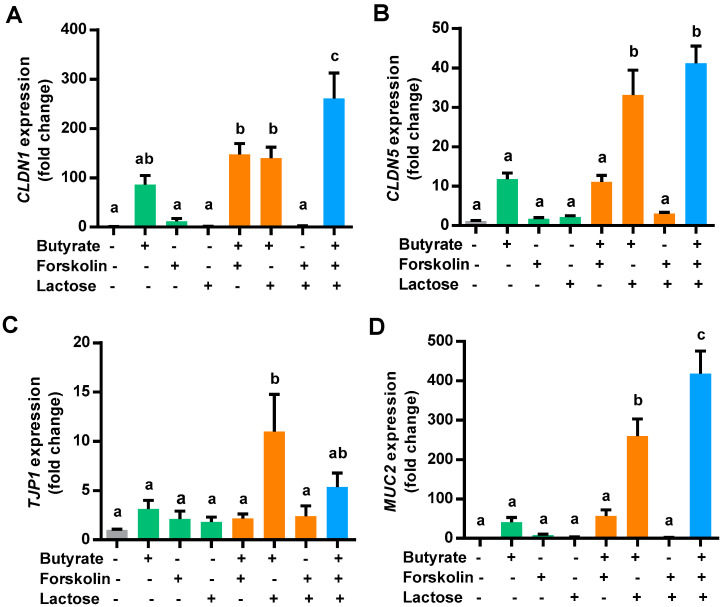
Induction of major barrier function genes by butyrate, forskolin, and lactose. Chicken HD11 cells were treated in triplicate with 2 mM butyrate, 5 μM forskolin, and 0.1 M lactose alone or in different combinations for 24 h, followed by RT-qPCR analysis of the expressions of claudin 1 (*CLDN1*) (**A**), *CLDN5* (**B**), tight junction protein 1 (*TJP1*) (**C**), and mucin 2 (*MUC2*) (**D**). Results are presented as means ± SEM of three independent experiments. Means not sharing a common letter are significantly different (*p* < 0.05; one-way ANOVA and *post hoc* Tukey’s test).

To examine a possible synergy among butyrate, forskolin, and lactose in improving barrier function, the gene expression levels of claudin 1 *(CLDN1), CLDN5*, tight junction protein 1 (*TJP1*)*,* and mucin 2 (*MUC2*) were quantified in chicken HD11 cells treated with the three compounds separately or in combinations. Consistently, 2 mM butyrate obviously induced all four barrier function genes examined, while 5 μM forskolin or 0.1 M lactose had only a marginal impact (Figure 3). A combination of butyrate and forskolin showed a modest 1.5-fold increase in inducing *CLDN1* relative to butyrate alone (Figure 3A), but no synergy was observed with *CLDN5* (Figure 3B), *TJP1* (Figure 3C), or *MUC2* (Figure 3D). On the other hand, butyrate was obviously synergistic with lactose in inducing all four barrier function genes. Desirably, the greatest synergy in inducing *CLDN1*, *CLDN5*, and *MUC2* were observed when butyrate, forskolin, and lactose were used together, except for *TJP1* (Figure 3). Taken together, butyrate, forskolin, and lactose demonstrated a synergy in stimulating the expressions of major barrier function genes, suggesting a possible role in enhancing mucosal barrier function.

### 2.3. Alleviation of NE in Broiler Chickens by Butyrate, Forskolin, and Lactose

Because of a synergistic effect among butyrate, forskolin, and lactose in enhancing the expressions of HDP and barrier function genes, we sought to further investigate the efficacy of the three compounds in protecting chickens against NE in two separate trials. In the first trial, we fed chicks with butyrate, forskolin-containing *C. forskohlii* (CF) extract, or lactose separately or in different combinations, followed by induction of NE. While mocked-infected chickens were all apparently healthy, 50% infected chickens died on nonmedicated diet at the end of the trial on day 18, and none of the individual compounds or two-compound combinations improved animal survival (Table 1). Supplementation with a combination of three compounds increased the survival of animals by 25% (Table 1). Among all infected chickens that survived, body weight of nonmedicated chickens in the NE group was 26% lower than that of mock-infected control chickenss, while the three-compound combination group tended to give the best numerical outcome, although statistically insignificant (Table 1). These results suggested a synergy among butyrate, forskolin, and lactose in protecting chickens from NE.

**Figure 4:**
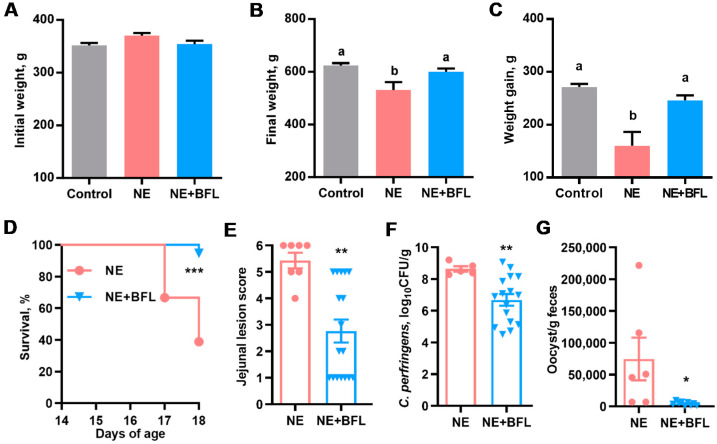
Alleviation of necrotic enteritis (NE) in broiler chickens by supplementation of butyrate, forskolin, and lactose. Day-old male Cobb chicks were supplemented with or without a mixture of 1 g/kg microencapsulated sodium butyrate, 10 mg/kg forskolin-containing *C. forskohlii* extract, and 10 g/kg lactose (NE + BFL) (*n* = 18) upon arrival. Chickens in the NE or NE + BFL group were orally challenged with 1 × 10^4^ *E. maxima* oocysts on day 11, followed by daily challenges with approximately 2 × 10^9^ CFU *C. perfringens* from days 14–16, while the animals in the Control group were mock-infected. Chickens were weighed individually on day 14 (**A**) and day 18 (**B**), and weight gain from days 14 to 18 (**C**) was calculated. Weight and weight gain were analyzed with ANOVA and *post hoc* Tukey’s test. Means with different letters are significantly different (*p* < 0.05). Animal survival from days 14–18 (**D**) was recorded daily and compared using the logrank test. On day 18, jejunal lesion score (**E**), jejunal *C. perfringens* titer (**F**), and oocyst shedding in the feces (**G**) were evaluated and subjected to unpaired Student’s *t*-test. Results are presented as means ± SEM. * *p* < 0.05, ** *p* < 0.01, and *** *p* < 0.001.

To confirm the protective effect of the three-compound combination against NE, a second trial was conducted with a larger number of animals per treatment group, without including individual compounds or any two-compound combinations. As expected, supplementation of the three compounds for the first 14 days had no effect on body weight (*p* = 0.17) (Figure 4A). NE caused a significant decline in body weight (*p* < 0.05), which was largely restored by butyrate, forskolin, and lactose (Figure 4B). The infection led to a marked weight loss during days 14–18, while feeding butyrate/forskolin/lactose counteracted the weight loss (*p* < 0.05) (Figure 4C). Moreover, the three-compound combination dramatically improved the survival of NE-afflicted chickens from 38.9% to 94.4% (*p* < 0.001) (Figure 4D). In fact, only one out of 18 chickens died in the three-compound combination group, while 11 chickens died or were euthanized in the nonmedicated group. Among surviving animals, NE caused severe lesions in the jejunum with an average score of 5.2 in a 6-point scoring system [25], while supplementation with the three compounds led to a significant reduction in the jejunal lesions to an average score of 2.8 (*p* < 0.01) (Figure 4E). As expected, mock-infected chickens had healthy intestines and were given a score of 0. Consistently, three compounds led to an approximately 100-fold reduction in the *C. perfringens* titer in the jejunum (*p* < 0.01) (Figure 4F). Additionally, the *E. maxima* oocyst shedding from the feces was significantly reduced by 17-fold in response to the three compounds (*p* = 0.02) (Figure 4G), while *E. maxima* oocysts were not observed in feces of healthy chickens in the control group. Taken together, supplementation of butyrate, forskolin, and lactose provides significant protection of animals against NE.

### 2.4. Attenuation of Coccidiosis in Broiler Chickens by Butyrate, Forskolin, and Lactose

**Figure 5:**
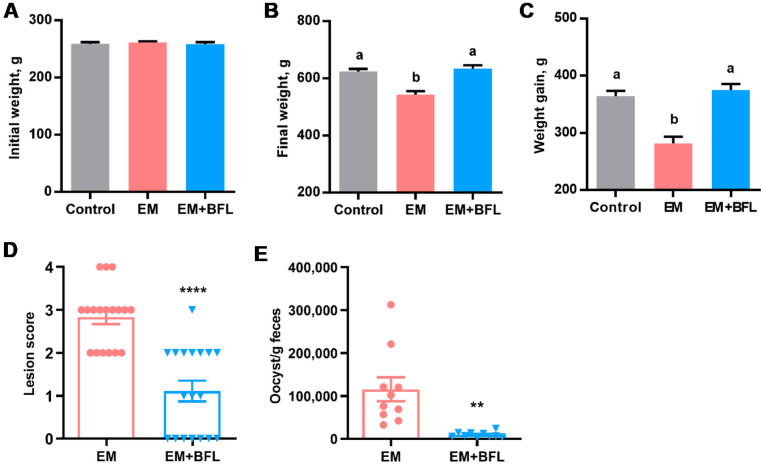
Attenuation of coccidiosis in broiler chickens by supplementation of butyrate, forskolin, and lactose. Day-of-hatch male Cobb chicks were supplemented with or without 1 g/kg microencapsulated sodium butyrate, 10 mg/kg forskolin-containing *C. forskohlii* extract, and 10 g/kg lactose (BFL) (*n* = 18) upon arrival. Animals in the EM or EM + BFL group were orally challenged with 2 × 10^4^ sporulated *E. maxima* (EM) on day 11 after overnight fasting, while the control group were mock-infected. Chickens were weighed individually on day 11 (**A**) and day 18 (**B**), and weight gain between days 11–18 (**C**) was calculated. Weight and weight gain were analyzed with one-way ANOVA and *post hoc* Tukey’s test. Means with different letters are significantly different (*p* < 0.05). Jejunal lesions (**D**) and fecal oocyst shedding (**E**) were examined on day 18 and subjected to unpaired Student’s *t*-test. Results are presented as means ± SEM. ** *p* < 0.01, and **** *p* < 0.0001.

A significant reduction in the fecal oocyst shedding in response to butyrate, forskolin, and lactose in the co-infection model of NE suggested the potential for the three compounds to directly mitigate coccidiosis. Therefore, a chicken model of coccidiosis [26,27] was employed for confirmation. As expected, the three compounds had no influence on body weight on day 11 (*p* = 0.79) (Figure 5A). *Eimeria* infection caused a 13% weight loss seven days post-infection (*p* < 0.05) (Figure 5B). Desirably, weight gain of *E. maxima*-challenged chickens was completely restored to the healthy level by the three compounds (Figure 5C). All mock-infected chickens in the control group had healthy intestines receiving a score of 0; however, *E. maxima* challenge caused obvious lesions in the jejunum with an average score of 2.8 in a 4-point scoring system [28], while the three compounds markedly decreased the lesion score to approximately 1.0 (*p* < 0.0001) (Figure 5D). Unsurprisingly, the three-compound combination also dramatically reduced the fecal oocyst shedding by approximately 12-fold seven days post-infection (*p* = 0.001) (Figure 5E). As expected, no oocysts were detected in the feces of mock-infected chickens. Altogether, dietary supplementation of butyrate, forskolin, and lactose confers significant protection of chickens from coccidiosis.

## 3. Discussion

The growing threat of antimicrobial resistance underlines a need for the development of effective alternatives to antibiotics for livestock production [4,5,6]. With antimicrobial, immunomodulatory, and barrier-protective activities, HDPs are critical effectors of innate immunity to help fight off infections [11,12,13,14,15]. A deficiency in HDP production is associated with increase susceptibility to infectious diseases, while augmenting HDP synthesis confers protection [7,8,9,10]. Enhancing animal innate defense through promoting the synthesis of endogenous HDPs is being explored as an alternative approach to in-feed antibiotics for disease control and prevention [7,8,9,10].

Butyrate is a well-known HDP inducer in humans and livestock animals [7,8,9,10]. Forskolin, a phytochemical, induces HDP gene expression in both human and chicken cells [22,23]. Lactose is also capable of enhancing the expression of *LL-37*, a human HDP gene in colonic epithelial cells [24]. In this study, 2–4 mM butyrate obviously induced the expressions of multiple HDP genes; however, 5 µM forskolin or 0.1 M lactose had a minimum effect. The reason is mainly because forskolin and lactose were used at suboptimal HDP-inducing concentrations in order to achieve an optimal synergy with butyrate. When used alone, 10 µM forskolin [23] and 0.2 M lactose [24] offer the peak HDP-inducing response, although at much lower magnitudes of induction than butyrate. We chose not to conduct the time-course experiments is due to the fact that butyrate [19], forskolin [23], and lactose [24] all give the peak HDP induction individually at 24 h.

Butyrate in combination with forskolin or lactose has induced higher levels of HDP expression relative to butyrate alone, consistent with earlier observations on the synergy between butyrate and forskolin [23] and between butyrate and lactose [24]. Here, we report for the first time the HDP-inducing synergy among butyrate, forskolin, and lactose. More importantly, the in vitro cell culture results have been extended in vivo. Supplementation of the three natural compounds offers significant protection of chickens from both severe NE and coccidiosis. Although several combinations of two HDP-inducing compounds, such as butyrate/forskolin [23], butyrate/lactose [24], butyrate/vitamin D_3_ [29,30], butyrate/wortmannin [31], and andrographolide/isoliquiritigenin [32], have been shown a synergy in HDP induction, this is the first report on the HDP-inducing synergistic effect among three small-molecule compounds.

However, the mechanism underlining the synergy in HDP induction among butyrate, forskolin, and lactose remains to be studied. Butyrate is known to modulate HDP expression by acting as a histone deacetylase inhibitor (HDACi) and mitogen-activated protein kinases (MAPK) signaling pathways are also involved [20,33]. In fact, a number of natural and synthetic HDACi have been identified to be capable of inducing HDPs [10,34]. Forskolin is a natural agonist of adenylate cyclase and activator of cyclic adenosine monophosphate (cAMP) signaling [21,35] and triggers HDP expression by activating cAMP signaling [22]. MAPK pathways are involved in forskolin-induced HDP expression [23]. Lactose also utilizes p38 and JNK MAPK pathway to upregulate HDP gene expression [24]. We have found that the synergy in HDP induction between butyrate and lactose is associated with an increase in histone acetylation and involves NF-κB, MAPK, and cAMP signaling pathways (unpublished results). Although histone acetylation, MAPK, cAMP, and NF-κB are all likely to participate in the HDP-inducing synergy among butyrate, forskolin, and lactose, their involvement needs to be experimentally verified.

Intestinal barrier function relies mainly on the mucus layer and tight junctions to defend against external insults [36,37]. MUC2 is a predominant constituent of the intestinal mucus, while multiple proteins such as claudins are involved in the assembly of tight junctions [36,37]. Consistent with its well-known barrier protective function [17,18], butyrate has been shown to induce the expressions of *MUC2* and tight junction proteins in this study. In addition, butyrate and lactose synergize with each other in *MUC2* and tight junction protein gene expression, and the strongest synergy occurs when butyrate, forskolin, and lactose are combined. Therefore, butyrate/forskolin/lactose-mediated protection of chickens from NE and coccidiosis is also likely attributed to enhanced intestinal barrier integrity beyond HDP induction. In fact, a few HDPs have also been shown to be capable of stimulating the synthesis of mucins and tight junction proteins [15]. HDPs induced by butyrate, forskolin, and lactose may also in turn promote mucosal barrier function. Furthermore, MUC2 was recently shown to induce human LL-37 synthesis [38], suggesting a positive feedback loop between HDPs and mucins. Collectively, mucosal barrier function is strengthened directly and indirectly by butyrate, forskolin, and lactose to help fight off infections.

Therefore, multiple mechanisms are likely involved in significant protection of animals against both bacterial and parasitic diseases observed with butyrate, forskolin, and lactose in this study. First, increased HDP synthesis in the intestinal tract is expected to result in enhanced killing of *C. perfringens* or *E. maxima* due to direct antibacterial and antiparasitic activities of HDPs [11,12] and indirect recruitment and activation of different types of immune cells [12]. Secondly, synergistic improvement in the barrier function will resist the colonization of *E. maxima* and *C. perfringens* in the intestinal tract. Thirdly, butyrate, forskolin, and lactose are all known to be anti-inflammatory [18,39,40], which is expected to alleviate the inflammatory response and hence facilitate the recovery in response to infections triggered by *E. maxima* and *C. perfringens*.

It is noted that significant protection of chickens from NE and coccidiosis was achieved by dietary supplementation of 1 g/kg microencapsulated sodium butyrate, 10 mg/kg 20% forskolin-containing *C. forskohlii* extract, and 10 g/kg lactose. Apparently, the concentration of each nature product needs to be further tested in order to achieve an optimal synergy in live animals. Moreover, many of the structural and functional analogs of butyrate such as short-chain fatty acids and their chemical derivatives and HDACi have been identified as potent HDP inducers as well [34,41,42,43]. Functional analogs of forskolin such as 8-bromo-cAMP, cholera toxin, and pertussis toxin are also capable of inducing HDP expression [23]. In addition to lactose, other sugars such as glucose, galactose, trehalose, and maltose enhance HDP expression as well [24]. Therefore, it is expected that a combination of the derivatives of butyrate, forskolin, and lactose will also be synergistic in HDP induction. Systematic screenings of different combinations of these derivatives could potentially yield more potent HDP inducers than butyrate, forskolin, and lactose per se to confer better protection against infectious diseases. Taken together, the synergy among butyrate, lactose, and forskolin or among their derivatives warrants further investigation for their potential as alternatives to antibiotic for control and prevention of various infections in not only poultry, but also other livestock species and even humans.

## 4. Materials and Methods

### 4.1. Culture and Stimulation of Chicken Macrophages and Jejunal Explants

Chicken HD11 macrophage cells [19,23] were cultured in complete RPMI 1640 (HyClone, Logan, UT, USA) containing 10% fetal bovine serum (Atlanta Biologicals, Flowery Branch, GA, USA), 100 U/mL penicillin, and 100 μg/mL streptomycin (Lonza, Walkersville, MD, USA) at 1 × 10^6^ cells/well in 12-well culture plates overnight, followed by treatment in triplicate with 2 mM sodium butyrate [19,23] (MilliporeSigma, St. Louis, MO, USA), 5 μM forskolin [23] (Santa Cruz Biotechnology, Dallas, TX, USA), or 0.1 M lactose (Thermo Fisher Scientific, Nazareth, PA, USA) separately or in combination. After incubation at 37 °C and 5% CO_2_ for 24 h, cells were lysed with RNAzol RT (Molecular Research Center, Cincinnati, OH, USA) for total RNA extraction according to the manufacturer’s protocol.

Chicken jejunal explants were prepared and cultured as previously described [23,31]. Briefly, the jejunal segments were collected from one- to two-week-old chickens, cultured in 6-well plates, treated in triplicate with 4 mM sodium butyrate [23], 5 μM forskolin [23], and 0.1 M lactose alone or in combination, and incubated in Hypoxia Chamber (StemCell Technologies, Vancouver, BC, Canada) filled with 95% O_2_ and 5% CO_2_ at 37 °C for 24 h, followed by centrifugation and RNA extraction.

### 4.2. RT-qPCR Analysis of Gene Expression

Following total RNA isolation from chicken HD11 cells or jejunal explants, RNA was quantified using Nanodrop 1000 (Thermo Fisher Scientific, Nazareth, PA, USA), followed by reverse transcription using iScript cDNA Synthesis Kit (Bio-Rad, Hercules, CA, USA) according to the manufacturer’s instructions. Quantitative PCR (qPCR) was performed with iTaq Universal SYBR Green Supermix (Bio-Rad, Hercules, CA, USA) and gene-specific primers (Table 2) as described [19,23]. The qPCR reactions were performed using CFX96 Real-time PCR Detection System (Bio-Rad, Hercules, CA, USA) with an initial activation at 95 °C for 10 min, followed by 40 cycles at 94 °C or 15 s, 60 °C for 20 s, and 72 °C for 30 s. The melting curves were analyzed to confirm the specificity of PCR amplifications. Gene expression levels were calculated with the 2^−^^ΔΔCt^ method using glyceraldehyde 3-phosphate dehydrogenase (*GAPDH*) as the reference gene.

### 4.3. Chicken NE Trials

Two NE trials were conducted to investigate the efficacy of butyrate, forskolin, and lactose in prevention of NE using a co-infection model as previously described [25,44]. All animal procedures were approved by the Institutional Animal Care and Use Committee of Oklahoma State University under Protocol number AG-16-10.

In the first trial, a total of 180 day-of-hatch male Cobb chicks were obtained from a commercial hatchery (Cobb-Vantress, Siloam Springs, AR, USA) and housed in an environmentally controlled room under standard management as recommended by Cobb-Vantress. Chickens were randomly distributed to one of nine dietary treatments with two floor pens per treatment and 10 birds per pen. Animals were given a standard soybean-corn mash starter diet (containing 21.5% crude protein) supplemented with or without 1 g/kg microencapsulated sodium butyrate (King Techina, Hangzhou, China), 10 mg/kg 20% forskolin-containing *C. forskohlii* (CF) extract (PureBulk, Roseburg, OR, USA), and 10 g/kg lactose (PureBulk, Roseburg, OR, USA) individually or in different combinations of 2–3 compounds. Animals had ad libitum access to respective diets upon arrival till the end of the trial.

After overnight fasting on day 10, six chickens were randomly selected from each pen and transferred to a battery cage on day 11 for infection with two cages per treatment (*n* = 12). Chickens in eight different group were orally challenged with 2 × 10^4^ sporulated *E. maxima* strain M6 oocysts (kindly provided by John R. Barta, University of Guelph, Canada) [27] in 1 mL saline on day 11, followed by daily challenges with approximately 8 × 10^8^ CFU of *C. perfringens* strain Brenda B carrying *netB* and *tpeL* toxin genes (kindly provided by Lisa Bielke at the Ohio State University, Columbus, OH, USA) [45] in 2 mL fluid thioglycollate (FTG) broth for four days from days 14 to 17. *C. perfringens* was cultured in chopped cooked meat medium and subsequently FTG for inoculation as described [25,44]. Animals in the control group were mock-infected with 1 mL saline and 2 mL FTG broth on days 11 and 14–17, respectively. Chickens were monitored twice daily throughout the experiment and those reluctant to move were euthanized by CO_2_ asphyxiation to minimize undue pain. Chickens were weighed individually on days 11 and 18 and weight gain was calculated. The survival of chickens was assessed on day 18.

A second NE trial was conducted to confirm the protection of chickens by a combination of butyrate, forskolin, and butyrate with a larger number of animals per treatment. A total of 90 day-old male Cobb chicks (Cobb-Vantress) were randomly allotted to one of three treatments (control, NE, and NE + BFL) with three floor pens/treatment and 10 chicks/pen. Similar to the first trial, chickens in the control and NE group were given a non-medicated basal starter diet (21.5% crude protein) upon arrival, while chickens in the NE+BFL group were supplemented with the mixture of 1 g/kg microencapsulated sodium butyrate, 10 mg/kg *C. forskohlii* extract, and 10 g/kg lactose (*n* = 18).

After overnight fasting on day 10, six chickens were randomly selected from each pen and transferred to a battery cage on day 11 for infection with three cages per treatment (*n* = 18). Chickens in the NE or NE + BFL group were orally challenged with 1 × 10^4^ *E. maxima* M6 oocysts on day 11, followed by daily challenges with approximately 2 × 10^9^ CFU *C. perfringens* for three days from days 14–16, while the animals in the control group were mock-infected. Chickens were monitored twice daily and those reluctant to move were euthanized to minimize undue suffering. Animals were weighed individually on days 14 and 18, and weight gain between days 14–18 was calculated. All surviving chickens were sacrificed on day 18 for intestinal lesion scoring and sample collection. The mid-jejunal content of each animal was aseptically collected and snap frozen at −80 °C for future quantification of *C. perfringens*. The jejunum was then cut open and scored for gross lesions of NE in a blind fashion using a 6-point scoring system [25]. Fresh fecal droppings from three animals in each cage were also collected separately (*n* = 9) and stored at 4 °C for counting of the oocysts.

### 4.4. C. perfringens Quantification

Microbial DNA was extracted from the jejunal digesta using ZR Fecal DNA MicroPrep Kit (Zymo Research, Irvine, CA, USA) and the concentration was measured with Nanodrop 1000. The *C. perfringens* titer in the digesta was quantified using a standard curve-based qPCR method [46]. A standard curve was established using serial 10-fold dilutions of pure *C. perfringens* genomic DNA with *C. perfringens*-specific primers [47]. The qPCR reactions were performed with an initial activation at 95 °C for 30 s, followed by 40 cycles at 94 °C for 5 s, 60 °C for 30 s, and 94 °C for 5 s. The *C. perfringens* titer was expressed as CFU per gram of the digesta.

### 4.5. Coccidiosis Trial

A total of 90 day-old male Cobb chicks were randomly allocated to one of three treatments (i.e., control, EM, and EM + BFL) with three floor pens/treatment and 10 chickens/pen. The control and EM groups were given a nonmedicated basal starter diet (21.5% crude protein), while chickens in the EM+BFL group were supplemented with the mixture of 1 g/kg microencapsulated sodium butyrate, 10 mg/kg *C. forskohlii* extract, and 10 g/kg lactose (*n* = 18) upon arrival. After overnight fasting on day 10, six chickens were randomly chosen from each pen and transferred to a battery cage on day 11, with three cages/treatment and six animals/cage (*n* = 18). Chickens in the EM and EM+BFL groups were orally gavaged with 2 × 10^4^ sporulated oocysts of *E. maxima* strain M6 in 1 mL saline, while the control group were mock-infected with 1 mL saline as described [26,27]. On day 18, chickens were euthanized by CO_2_ asphyxiation. Chickens were individually weighed on days 11 and 18, and weight gain was calculated. Jejunal lesions of coccidiosis were blindly examined using a 4-point scoring system [28]. Fresh fecal droppings from three animals in each cage (*n* = 9) were gathered separately (*n* = 9) and stored at 4 °C for counting of the oocysts.

### 4.6. Counting of the Oocyst Shedding in the Feces

The oocyst shedding in the feces was determined using a modified McMaster technique as described [48]. Briefly, approximately 0.5–1 g of the feces from each chicken were weighed and mixed with 30 volumes of Feca-Med Sodium Nitrate Premixed Fecal Flotation Medium (Allivet, Miami Lakes, FL, USA). The mixture was thoroughly vortexed on a vortex mixer and filtered through four layers of cheesecloth (Fisher Scientific, Hampton, NH, USA). The filtrate was then quickly pipetted into two chambers of a McMaster slide (Chalex, Park City, UT, USA). After standing for 5 min, the floating oocysts within the gridded area of both chambers were counted under a microscope at 100× magnification. The oocyst counts per gram of feces was calculated as 10× the total number of oocysts counted in both chambers.

### 4.7. Statistical Analysis

Data analysis and visualization were implemented in Prism (GraphPad, La Jolla, CA, USA). Results were presented as means ± standard error of the mean (SEM). The survival rate among different treatments was analyzed using the logrank test. Other parameters were compared among treatments using one-way ANOVA and *post hoc* Tukey’s test (if more than two treatments were involved), or unpaired Student’s *t*-test (if only two treatments were involved). Statistical significance was considered if *p* < 0.05.

## 5. Conclusions

In summary, butyrate, forskolin, and lactose shows a strong synergy in improving HDP expression in vitro and ex vivo. Barrier function genes were also induced in chicken cells by the three natural products. Dietary supplementation of sodium butyrate, forskolin-containing plant extract, and lactose dramatically reduced the lethality, weight loss, intestinal lesions, and pathogen colonization in both NE and coccidiosis. A mixture of butyrate, forskolin, and lactose or their derivatives may be further explored as a promising alternative to antibiotics for disease control and prevention in poultry and possibly other animal species as well.

## Figures and Tables

**Table 1:** Survival rate and weight gain of chickens with necrotic enteritis ^1^.

Variables	Control	NE	B	F	L	BF	BL	FL	BFL	SEM	*p*-Value ^2^
Survival rate,%	100 (12/12)	50.0 (6/12)	50.0 (6/12)	25.0 (3/12)	41.7 (5/12)	41.7 (5/12)	41.7 (5/12)	33.3 (4/12)	75.0 (9/12)		0.018
Initial weight, g	303.7	299.2	300.8	303.3	296.8	299.2	295.0	308.3	304.3	7.78	0.98
Final weight, g	665.3 ^a^	490.2 ^b^	543.2 ^b^	556.7 ^b^	512.0 ^b^	521.2 ^b^	545.2 ^b^	508.0 ^b^	560.7 ^b^	20.23	<0.0001
Weight gain, g	361.7 ^a^	191.0 ^b^	242.3 ^b^	253.3 ^b^	215.2 ^b^	222.0 ^b^	250.2 ^b^	199.8 ^b^	256.3 ^b^	19.91	<0.0001

^1^ Day-of-hatch male Cobb broiler chicks were randomly divided into nine groups (*n* = 12) and supplemented with or without 1 g/kg microencapsulated sodium butyrate (B), 10 mg/kg of 20% forskolin-containing *Coleus forskohlii* extract (F), and 10 g/kg lactose (L) individually or in different combinations (BF, BL, FL, and BFL) from day-of-hatch till the end of the trial. All animals were orally inoculated with 2 × 10^4^ sporulated *E. maxima* oocysts on day 11, followed by daily challenges with approximately 8 × 10^8^ CFU *C. perfringens* for four days from day 14 to 17, except for the animals in the control group were mock-infected only with the medium each time. Animals in the NE group were infected, but received no medication in the diet. The survival of chickens was assessed on day 18. Chickens were weighed individually on days 11 and 18 and weight gain was calculated. ^2^ Statistical significance on the survival rate was determined using the logrank test, while body weight and weight gain were determined using one-way ANOVA and *post hoc* Tukey’s test. The values in a row not sharing a common letter are significantly different (*p* < 0.05).

**Table 2:** Sequences of primers used in RT-qPCR.

Gene ^1^	Forward Primer (5′ to 3′)	Reverse Primer (5′ to 3′)	Product Size, bp	GenBank Accession Number ^2^
*A* *v* *BD3*	ATGCGGATCGTGTACCTGCTC	CAGAATTCAGGGCATCAACCTC	196	NM_204650.2
*A* *v* *BD8*	TTCTCCTCACTGTGCTCCAA	AAGGCTCTGGTATGGAGGTG	124	NM_001001781.1
*A* *v* *BD9*	GCAAAGGCTATTCCACAGCAG	AGCATTTCAGCTTCCCACCAC	211	NM_001001611.2
*A* *v* *BD10*	TGGGGCACGCAGTCCACAAC	ATCAGCTCCTCAAGGCAGTG	298	NM_001001609.2
*CLDN1*	TTCCAACCAGGCTTTATGATG	TGCAGAGTCAGGTCAAACAGA	140	NM_001013611.2
*CLDN5*	CATCACTTCTCCTTCGTCAGC	ATCTCCCAGGTCTCTGCATTT	103	NM_204201.1
*TJP1*	CATCAGCCAGAAGAGAACCAG	CCAAGAACAAAAGTGGTATGC	117	XM_037393868.1
*MUC2*	TCTGGAGAGAGTTGTCCTGAC	TCCTTGCAGCAGGAACAACT	105	XM_021402134.1
*GAPDH*	GCACGCCATCACTATCTTCC	CATCCACCGTCTTCTGTGTG	356	NM_204305.1

^1^ Abbreviations used: *AvBD*: avian β-defensin; *CLDN1*: claudin 1; *CLDN5*: claudin 5; *TJP1*, tight junction protein 1; *MUC2*: mucin 2. ^2^ Primers for *AvBD3*, *AvBD8*, *AvBD9*, *AvBD10*, and *GAPDH* were adopted from reference [19].

## Data Availability

All data generated during this study are included in this published article.
